# Application and evaluation of nanopore targeted sequencing in the detection of bacterial and fungal pathogens in leukemia bloodstream infections

**DOI:** 10.3389/fmicb.2026.1866353

**Published:** 2026-09-24

**Authors:** Qian Yao, Ling Tang, Daozi Jiang, Ming Wang

**Affiliations:** 1 Department of Clinical Laboratory, Renmin Hospital of Wuhan University, Wuhan, China; 2 Hubei Jiangxia Laboratory, Wuhan, China; 3 Department of Hematology, Renmin Hospital of Wuhan University, Wuhan, China

**Keywords:** application, bloodstream infection, leukemia, nanopore targeted sequencing, pathogen identification

## Abstract

**Objective:**

To evaluate the diagnostic performance and clinical impact of microbial nanopore-targeted sequencing (NTS) technology on pathogenic microorganisms in leukemia patients.

**Methods:**

In this retrospective observational study, we recruited 206 patients to study the effectiveness of NTS in the identification of infectious pathogens. The detection performance of NTS was compared with that of blood culture (BC).

**Results:**

The positive rate of NTS was significantly higher than that of BC [62.62% (129/206 cases) vs. 3.88% (8/206 cases)]. Compared with BC, the positive and negative percentage agreement of NTS were 87.50% (95%CI: 47.35–99.68%), (7 of 8 cases) and 38.38% (95%CI: 31.52–45.60%), (76 of 198 cases), respectively. The sensitivity of NTS for diagnosing bloodstream infections was 65.32% (95%CI: 56.21–73.69%), (81/124), which was significantly higher than BC [6.45% (95%CI: 2.84–12.13%), (8/124)], *P* < 0.001. The specificity of NTS was 41.46% (95%CI: 30.17–53.52%%), (34/82), and that of BC was [100%(95%CI: 95.56–100.00%) (82/82)]. The medication of 32 participants (24.81% [32/129]) was modified by NTS results. Among 206 participants, 29 cases who adjusted their medication based on NTS results showed improved clinical outcomes.

**Conclusion:**

NTS increases pathogen-detection yield but has low specificity and can be used as an adjunctive diagnostic method for leukemia BSI.

## 1 Introduction

Leukemia is a common malignant tumor of the hematopoietic system in clinical practice. The disease is mainly caused by abnormal proliferation of leukemia cells in hematopoietic stem cells, which seriously affects the normal operation of the hematopoietic system and ultimately leads to damage to various tissues and organs (Chen et al., 2023). In recent years, with the continuous advancement of medical technology, the treatment methods for hematological malignancies have also been constantly updated. However, as one of the common complications in patients with hematological malignancies, the diagnosis and treatment of bloodstream infections still face many challenges. Infectious shock is common in patients with hematological malignancies and carries a high risk of mortality (Society of Critical Care Medicine and Chinese Medical Association, 2007). In addition, patients receiving immunosuppressive drugs for T cell function, as well as those undergoing newly developed targeted molecular therapies, are at risk of more complex pathogen infections (such as viruses, bacteria, fungi, and parasites) (Chamilos et al., 2018; Marchesi et al., 2018; Society of Critical Care Medicine and Chinese Medical Association, 2007). Previous studies have reported that among immunocompromised patients with hematologic diseases who develop bloodstream infections, the most common bacterial, viral, and fungal pathogens are *Escherichia coli*, Candida species, and Epstein-Barr virus (Hong et al., 2023). Long-term use of broad-spectrum antibiotics can increase the risk of infection by multidrug-resistant bacteria, and early downgrading of antibiotics has a significant impact on the microbial ecology that relies on pathogen identification. Although traditional blood culture (BC) methods can help doctors determine whether patients have bloodstream infections to some extent, their detection cycle is long and sensitivity is limited, resulting in less than 15% of infected patients being recorded through BC (Rapszky et al., 2025). Since bloodstream infections in immunodeficient leukemia patients can rapidly progress to respiratory and circulatory failure, they must be treated as medical emergencies necessitating prompt antibiotic intervention. Traditional microbiological methodologies such as blood culture or matrix assisted laser desorption ionization time of flight mass spectrometry (MALDI-TOF MS) rely on blood culture enrichment limited by poor sensitivity, narrow detection ranges, or prolonged turnaround times in the diagnosis of bloodstream infections (Florio et al., 2018). Therefore, there is an urgent need for more rapid and sensitive detection technologies. As a new molecular biological technology, nanopore targeted sequencing (NTS) has attracted increasing attention in the diagnosis of bloodstream infection (Carandang et al., 2025; Cunanan et al., 2026; Sheka et al., 2021). NTS technology offers a sensitive and comprehensive approach to pathogen detection, covering not only rare bacteria such as *Chryseobacterium indologenes*, etc, but also special pathogens, including fungi and *Mycobacterium* species (Hong et al., 2023). These pathogens can lead to active infections that may prove fatal in affected patients with Immunodeficiency (Hong et al., 2023). The advantage of rapid diagnosis is considered to bring great help to patients with hematological malignancies. The diagnostic and antimicrobial treatment guidance value of NTS in patients with leukemia bloodstream infection is currently unclear.

The purpose of this study was to compare the detection results of NTS and conventional methods in patients with hematological disease clinically diagnosed as bloodstream infection through a retrospective, single-center diagnostic observational study, and to evaluate the ability of NTS as a pathogen identification in patients with clinical hematological disease complicated with bloodstream infection. All data were collected from existing clinical records. We aimed to evaluate the diagnostic performance and clinical impact of NTS detection compared with BC and clinical diagnosis in real time, to provide a more reliable and rapid diagnostic method for the clinic.

## 2 Materials and methods

### 2.1 Research design

From November 2021 to October 2023, a retrospective, single-center diagnostic observational study was conducted to evaluate the impact of NTS and BC on clinical judgment and whether the results of NTS caused changes in antimicrobial therapy in leukemia patients and promoted the effect of antimicrobial therapy. The scheme was designed by the author and approved by the ethics committee of Renmin Hospital of Wuhan University (WDRY2025-K200). Blood samples were taken for NTS sequencing, and blood culture was performed at the same time in Renmin Hospital of Wuhan University. The clinical and experimental data of the patients were recorded. The main purpose of this study was to evaluate the diagnostic performance of NTS, and the secondary purpose was to evaluate the clinical application value of NTS in leukemia patients with blood infection.

### 2.2 Study population

The subjects were leukemia inpatients in the Department of Hematology, Renmin Hospital of Wuhan University. The inclusion criteria were as follows: patients diagnosed with leukemia through hematological examination and bone marrow analysis, including acute myeloid leukemia (AML), chronic myeloid leukemia (CML), acute lymphoblastic leukemia (ALL), and chronic lymphocytic leukemia (CLL), who presented with evident signs of infection. Each patient contributed only one infectious episode to the study. At the time of infection, blood samples were collected from the patients for blood culture, pathogen nucleic acid testing, high-throughput sequencing, and other examinations. The exclusion criteria were: (1) combined with other serious basic diseases (such as heart, liver, renal failure, etc.) or active severe infections (such as AIDS, etc.), which may interfere with the study results. (2) patients with previously confirmed autoimmune diseases. The diagnosis of bloodstream infection relies primarily on the recognition of clinical symptoms, followed by confirmation through laboratory testing. Obvious symptoms and signs of infection were as follows (Ministry of Health of the People’s Republic of China, 2001): Systemic infectious symptoms: The patient presents with fever (body temperature > 38°C) or hypothermia (<36°C), often accompanied by chills, tachycardia, tachypnea, hypotension (systolic blood pressure < 90 mmHg or a decrease of > 40 mmHg from baseline), confusion, organ dysfunction, and other signs of systemic toxicity. Local or associated findings: there is evidence of a clear portal of infection (e.g., intravenous catheter, surgical wound, skin and soft tissue infection, etc.) or metastatic infectious foci; alternatively, the patient may present with rash, petechiae, hepatosplenomegaly, leukocytosis with a left shift, elevated C-reactive protein, and elevated procalcitonin, with no other obvious cause identified. Exclusion of other conditions: Non-infectious causes of fever (e.g., drug fever) and systemic inflammatory responses that cannot be fully explained by infection at other sites must be ruled out. A positive blood culture remains the gold standard for the microbiological diagnosis of bloodstream infection. However, as blood culture results are affected by various factors, a negative blood culture cannot exclude the possibility of bloodstream infection. Two infectious disease attending physicians independently evaluated each case in accordance with predefined standardized bloodstream infection diagnostic criteria and performed a composite adjudication of clinical diagnosis, clinical antimicrobial efficacy and treatment rationality for every patient based on symptoms and signs, radiological findings, infection-related laboratory examinations and microbiological test results, with any inter-reviewer disagreements settled through third-party arbitration by a senior chief physician. The diagnosis of bloodstream infection requires comprehensive consideration of the above indicators. Although adjudicators are not completely blinded to the NTS results, these NTS results are not the primary indicators for clinical diagnosis of bloodstream infections. Instead, NTS results are more often used as an auxiliary verification method for confirmed bloodstream infections, as well as a guiding indicator for adjusting antibiotics.

In terms of the selection, adjustment and rationality of the antimicrobial regimen, we followed the Chinese Guidelines for the Treatment of Severe Sepsis/Septic Shock (2014), Guidelines for Prevention and Treatment of Intravascular Catheter Associated Infections (2007), Guidelines for Hemodynamic Monitoring and Support of Severe Infection and septic shock in Adults (2006), and Chinese guidelines for the clinical application of antibacterial drugs for agranulocytosis with fever (2016) (Chinese Society of Hematology, Chinese Medical Association, and Chinese Medical Doctor Association, Hematology Branch, 2016; Society of Critical Care Medicine and Chinese Medical Association, 2015; Society of Critical Care Medicine and Chinese Medical Association, 2008; Society of Critical Care Medicine and Chinese Medical Association, 2007). Clinical Practice Guideline for the Use of Antimicrobial Agents in Neutropenia Patients with Cancer (Freifeld et al., 2011). Clinicians prescribe the initial empirical antimicrobial treatments (IEATs) mainly according to clinical symptoms, infection sites, primary lesions, previous infections, and the risk of drug-resistant bacteria colonization (Rhodes et al., 2017). For febrile immunocompromised patients with leukemia, intravenous empirical antibiotic monotherapy with an antipseudomonal β-lactam agent [e.g., cefepime, a carbapenem (meropenem or imipenem-cilastatin), or piperacillin-tazobactam] is recommended. Other agents, such as aminoglycosides, fluoroquinolones, and/or vancomycin, may be added to the initial regimen in the presence of hypotension or when antimicrobial resistance is suspected. For patients with prolonged immunocompromised status who remain febrile despite the above antibiotic therapy for 4–7 days, the addition of antifungal agents may also be considered. Within 24 h after the pathogen results from NTS, blood culture, and antimicrobial susceptibility testing based on blood culture become available, antibiotic therapy should be adjusted promptly based on the identified pathogen and its susceptibility profile. The NTS test results we obtained are the pathogen detection results (bacteria or fungi) after excluding contamination during collection or packaging, as well as laboratory contamination. These results are then carefully interpreted and evaluated in conjunction with the level of pathogen DNA reads, clinical symptoms, and other laboratory test results. Clinical symptoms and laboratory tests include: the patient still has repeated fever, systemic toxic symptoms, rashes, bleeding symptoms, etc. not improving, and laboratory infection indicators C-reactive protein (CRP), procalcitonin (PCT) not decreasing. Whether the follow-up antimicrobial treatment plan will change is largely based on the effectiveness of IEAT, microbial test results (BC and NTS), and other routine examination results, including CRP, PCT, white blood cell count (WBC), neutrophil count (NEC), platelet count (PLT), and hemoglobin (Hb). Based on the patient’s body temperature, infection markers (such as CRP, PCT), and imaging improvement, the step-down treatment or further adjustment of medication was selected. Prior antibacterial treatment (PAT) indicates prior exposure to antibacterial drugs such as imipenem, cilastatin sodium, cefotiam hydrochloride, vancomycin hydrochloride, linezolid, moxifloxacin hydrochloride, cefathiamidine, etc. For cases with positive pathogens detected by BC and NTS, the treating doctor assesses whether the initial antibiotic treatment encompasses all detected pathogens and whether the blood culture and antimicrobial susceptibility test results accurately identify the therapeutic drugs, or if combination therapy is necessary. At the same time, verify that the detected pathogens align with the patients’ clinical symptoms and other routine detection indicators to adjust or maintain the medication plan as necessary.

### 2.3 Blood culture

The timing of blood collection should ideally precede antibiotic use as much as possible. Alternatively, it should coincide with nanopore sequencing blood collection. In either case, microbial culture should be performed on the patients’ blood samples. Use BD Bactec resin containing aerobic blood culture bottles and/or hemolysin-containing anaerobic blood culture bottles/children’s bottles. After removing the plastic cap from the mouth of the culture bottle, disinfect the rubber stopper of the blood culture bottle with 75% alcohol for 60 s. After it dries naturally, inject the specimen into the culture bottle and mix the specimen and culture medium upside down slightly to avoid blood agglutination. The ratio of blood volume to medium volume should be 1:5∼1:10. The blood volume of adults was 8–10 mL, and that of children was 3–5 mL. After the sample collection, the blood culture specimens were tested within 2 h. If the blood culture result is positive, inoculate the blood agar (BA) plate and Vancouver Agar (VA) and place them in a 35°C, 5% CO_2_ incubator for 72–120 h to observe whether there is microbial growth. If the microorganism grows, a Bruker matrix-assisted laser desorption time of flight mass spectrometer (MALDI-TOF/TOF) was used for rapid identification. At the same time, the corresponding drug sensitivity test was carried out.

### 2.4 Specimen collection and laboratory testing

Blood samples obtained according to standard procedures were cultured to identify bacteria and fungi. In addition, 5 mL of whole blood samples from each patient were placed in sterile tubes, and the mixture of plasma and leukocytes was taken for NTS analysis. Mixed infection was defined as the infection with two or more types of pathogen found at the same time (combined with the detection results of NTS and BC). At the same time, the levels of NEC, Hb, PLT, PCT, CRP, and WBC were detected.

### 2.5 Blood sample preparation and nucleic acid extraction

Turn the EDTA tube containing blood upside down, and mix well. Take 1 mL blood sample in a 1.5 mL EP tube. Centrifuge at low speed (50 × g, 10 min). After centrifugation, transfer the upper plasma and middle leukocyte layer to the grinding tube (avoid sucking the lower erythrocyte). The above grinding tube was centrifuged at high speed (12,000 rpm, 10 min), and the supernatant was discarded to a 300 μL volume of sediment for DNA extraction. Nucleic acid extraction: add 150 μL lysate 1, 150 μL lysate 2, and 30 μL nucleic acid protectant into the grinding tube containing the sample and vortex evenly. The mixed samples were mixed with TGrinder H24 tissue grinding homogenizer (OSE-TH-01) (3800 rpm/s speed oscillation for 30 s, interval for 30 s, a total of 2 cycles). After grinding, centrifuge (7,000 rpm, 3 min), add 20μL protease K into the grinding tube, vortex oscillation and mixing, and then place in the metal bath for incubation at 65 °C for 10 min. After incubation, shake slightly, mix evenly, and then centrifuge immediately. Take 550 μL of liquid for the next extraction. Nucleic acid purification: mix magnetic beads and nucleic acids of the same volume, shake and mix well, and then stand at room temperature for 10 min. After instantaneous centrifugation, put it on the magnetic frame and wait for 2 min until it is clear (if the solution is not clear, extend the magnetic absorption time until the solution is completely clear). Discard the supernatant, and avoid touching the magnetic beads. Add 750 μL of 80% ethanol for washing, wait for 30 s, discard the supernatant, and avoid touching the magnetic beads. Cover the EP tube, centrifuge instantaneously, and suck and discard the liquid at the bottom of the tube. Centrifuge instantaneously, drop the liquid on the wall, insert the EP tubes into the magnetic frame, and wait for 1 min. Open the tube cover, suck out the residual ethanol and discard it, and wait for 1 min at the opening (wait for the ethanol to volatilize completely). Add 41 μL of DNase/RNase-free H_2_O into the EP tube, shake and mix well, and then stand at room temperature for x min. After 35 s of instant separation, place it on the magnetic frame until it clears. Transfer 40 μL of nucleic acid into the new EP tube. Avoid attracting magnetic beads during the transfer process.

### 2.6 Nanopore targeted sequencing

NTS was an amplicon-based, targeted sequencing pathogen panel. All primers used in this study are following the previous study (Hong et al., 2023). The barcoded products of the 16S rRNA gene and ITS1/2 amplification from the same samples were pooled at a mass ratio of 10:3. The pooled products from the different samples were mixed with equal quality for one sequencing library, which prepared using the Nanopore Ligation Sequencing Kit (SQK-LSK109, Oxford Nanopore, Oxford, United Kingdom). The library was sequenced with an R9.4.1 flow cell (FLO-MIN106, Oxford Nanopore Technologies) by using Oxford Nanopore GridION X5. The bioinformatics method and pathogen determination are following the previous study (Hong et al., 2023). FAST5 files containing raw Nanopore signal data were base called and converted to FASTQ format in real-time using Guppy (v3.3.0). High-quality data were obtained by removing exhibited low-quality read (Q < 7) and read with undesired length ( < 200 nt or > 2,000 nt). Adapter and barcode sequences were trimed by using Porechop (v.0.2.4) from the high-quality data. After removing adapter and barcode sequences, the following reads were compared with the 16S rDNA/ITS database (ftp://ftp.ncbi.nlm.nih.gov/refseq/TargetedLoci) using BLASTn (v.2.9.0+). The taxon of the reads assigned to the same species was based on a consensus sequence was maked by Medaka (v.0.10.1) from the reads assigned to the same species and compared with the reference databases. The criteria used to select these pathogens as follows: (1) Filtering out closely related microorganisms (Gu et al., 2021). (2) Filtering out contaminants from negative controls (Jing et al., 2021). A list of clinical pathogens was used to identify the pathogens (Supplementary material; Blauwkamp et al., 2019). Organisms outside the list will be considered as fixed value bacteria or environmental bacteria. The final report for each sample was discussed individually with a clinical microbiology specialist and clinicians responsible for each patient.

### 2.7 Differentiation of pathogenic and background microorganisms via comprehensive clinical evidence

The high sequence number (high detection amount and high abundance) is consistent with the clinical symptoms: whether there are infection symptoms (fever, cough, etc.), the site of infection (blood, cerebrospinal fluid and other sterile body fluids with great positive significance), and other tests: whether the results are consistent with the culture and PCR, whether the inflammatory indicators (WBC, CRP, PCT) are increased, and the treatment response: after the treatment of the detected bacteria, the patient is improved to comprehensively determine that the organism has clinical significance and conduct corresponding treatment as a real blood stream pathogen. If a low sequence number of opportunistic pathogens is inconsistent with the patient’s infection symptoms, infection sites, and the results of other detection indicators, the bacteria may be determined as contaminated or background bacteria. It should be noted that some rare bacteria, even if NTS test results are low abundance, should also be paid attention to in patients with specific immune deficiency.

### 2.8 Statistical analysis

The diagnostic efficacy of NTS was evaluated by combining BC results and clinical adjudication. The composite clinical diagnosis was identified as the reference standard for diagnostic accuracy. According to the extracted data, a 2 × 2 contingency table was established, and the sensitivity (SN), specificity (SP), positive predictive value (PPV) and negative predictive value (NPV) of NTS and BC detection methods for disease detection were calculated and described based on the composite clinical diagnosis. At the same time, in order to intuitively compare and evaluate the NTS detection method with the traditional BC detection method, we use BC method as the microbiological comparator, and establish a 2 × 2 contingency table, which were described as referring to BC: “True positive” was defined as the positive NTS results with a positive BC result; “false positive” denoted a positive NTS result with a negative BC result; “false negative” was characterized by a negative NTS result with a positive BC result; when both NTS and BC results were negative, it was defined as “true negative.” Positive percentage agreement (PPA) represented the proportion of NTS-positive cases among BC-positive results, while negative percentage agreement (NPA) denoted the proportion of NTS-negative cases among BC-negative results. Improved clinical outcomes referred to the improvement of clinician-documented improvement made by independent clinicians for each patient based on symptoms and signs, radiological testing results, infection-related laboratory tests, and results of microbiological tests. The time of discharge was used as the evaluation window. Clinical improvement was judged by independent infectious disease specialists blinded to NTS data, separate from ward clinicians who adjusted antimicrobials. Mixed pathogens referred to bacterial, fungal, mycoplasma two or more simultaneous infections. Diagnostic performance analysis based on original sequencing results.

For baseline characteristics and common laboratory tests, continuous variables were described as median (interquartile range), and categorical variables were described as numbers (percentages). Chi-square test and Fisher’s exact test were used for group comparison. McNemar’s test for paired proportions was used to compare sensitivity and specificity of NTS and blood culture. 95% CI was estimated by the exact binomial method. *P* < 0.05 was considered statistically significant. The clinical medication judgment and therapeutic intervention effect of NTS and BC on patients with suspected infection was described using Sankey diagrams. SPSS 25.0 and GraphPad Prism 9 were used for data analysis^1^.

## 3 Results

### 3.1 Patient characteristics

During the period from November 2021 to October 2023, 246 participants were screened for review. 239 participants met the inclusion criteria, of which 12 blood samples did not pass the quality acceptance, 5 were enrolled without blood culture, the medication history records of 16 patients were incomplete, and finally 206 participants were enrolled and completed the study (Figure 1). Patient characteristics were summarized in Table 1. The median age was 57 years (16–84 years). Among 206 patients, 101 were male, accounting for 49.03%. The cohort mainly included patients with acute myeloid leukemia [166 of 206 cases (80.58%)], patients with chronic myeloid leukemia [7 of 206 cases (3.40%)], patients with acute lymphoblastic leukemia [19 of 206 cases (9.22%)], and patients with chronic lymphoblastic leukemia [14 of 206 cases (6.80%)]. A total of 8.25% (17 of 206 participants) had diabetes before recruitment, while 13.59% (28 of 206 participants) had hypertension. Among 206 participants, 88 (42.72%) used an indwelling venous catheter. 124 patients were diagnosed with bloodstream infection. The BC results of 8 patients were positive, and 198 patients were negative. The total mortality rate was 3.40% (7 of 206 cases), and all died within 28 days. In addition, we compared the routine laboratory test results, including NEC, Hb, PLT, PCT, CRP, and WBC, of patients with bloodstream infections and non-bloodstream infections undergoing nanopore sequencing testing, and found no significant differences, with the *P*-value were 0.329, 0.727, 0.465, 0.252, 0.139, and 0.963, respectively (Table 2).

**TABLE 1:** Demographic and clinical characteristics of the 206 patients.

Characteristic	Value
Age (y)	
Median (range)	56.5 (46–64)
Distribution, n(%)	
16–17	5 (2.43%)
18–45	44 (21.36%)
46–69	133 (64.56%)
70–84	24 (11.65%)
Male sex, n(%)	101 (49.03%)
Leukemia type, n(%)	
AML	166 (80.58%)
CML	7 (3.40%)
ALL	19 (9.22%)
CLL	14 (6.80%)
Diabetes, n(%)	
Yes	17 (8.25%)
No	190 (92.23%)
Hypertension, n(%)	
Yes	28 (13.59%)
No	178 (86.41%)
Venous catheter indwelling, n(%)	88 (42.72%)
Length of stay(range) (d)	4–59
Median in hospital	26 (4–59)
Death within 28 d, n(%)	7 (3.40%)
Clinical diagnosis	
Bloodstream infections, n(%)	124 (60.19%)
Non-bloodstream infections, n(%)	82 (39.81%)
BC positive, n(%)	8 (3.88%, 95%CI: 1.69–7.50%)
BC negative, n(%)	198 (96.12%, 95%CI: 92.50–98.31%)
Clinical outcome	
Improved, n(%)	179 (86.89%)
Not improved, n(%)	20 (9.71%)
Death, n(%)	7 (3.40%)

SD, Standard deviation; AML, Acute myeloid leukemia; CML, Chronic myelogenous leukemia; ALL, Acute lymphoblastic leukemia; CLL, Chronic lymphocyte leukemia; Improved: the improvement of clinician-documented improvement made by independent clinicians for each patient based on symptoms and signs, radiological testing results, infection-related laboratory tests, and results of microbiological tests; Not improved: unimproved clinical results recorded by the independent clinician for each patient according to the symptoms and signs, radiological testing results, infection-related laboratory tests, and results of microbiological tests.

**TABLE 2:** Clinical laboratory indicators between bloodstream infection group and non-bloodstream infection group.

Variable	Bloodstream infection (124)	Non-bloodstream infection (82)	*P*-value
Bacterial infection	71	–	–
Fungal infection	20	–	–
Mycoplasma infection	1	–	–
Mixed infection^a^	8	–	–
PAT, n(%)			
Yes	111 (89.52%)	72 (87.80%)	0.703
No	13 (10.48%)	10 (12.20%)	
NEC (*10^9^/L)	0.33 (0.06–2.65)	0.68 (0.07–2.86)	0.329
Hb (g/L)	67.50 (58.00–80.75)	67.00 (59.75–78.25)	0.727
PLT(*10^9^/L)	25.00 (15.00–54.00)	28.00 (15.00–76.75)	0.465
PCT (ng/mL)	0.16 (0.09–0.58)	0.14 (0.07–0.41)	0.252
CRP(mg/L)	91.08 (34.88–161.00)	67.37 (25.48–132.88)	0.139
WBC (*10^9^/L)	1.61 (0.45–6.72)	1.88 (0.57–5.26)	0.963

CRP, C-reactive protein; PLT, Platelets; Hb, Hemoglobin; PCT, Procalcitonin; WBC, white blood count; NEC, neutrophil count; PAT, prior antibacterial treatment: prior exposure to antibacterial drugs such as imipenem, cilastatin sodium, cefotiam hydrochloride, vancomycin hydrochloride, linezolid, moxifloxacin hydrochloride, cefathiamidine, etc.;

*^a^*Mixed pathogens: bacterial, fungal, mycoplasma two or more simultaneous infections, seven patients were defined as bacterial and fungal bloodstream co-infection, and one was defined as bacterial and mycoplasma bloodstream co-infection. *P* < 0.05, statistically significant.

**FIGURE 1:**
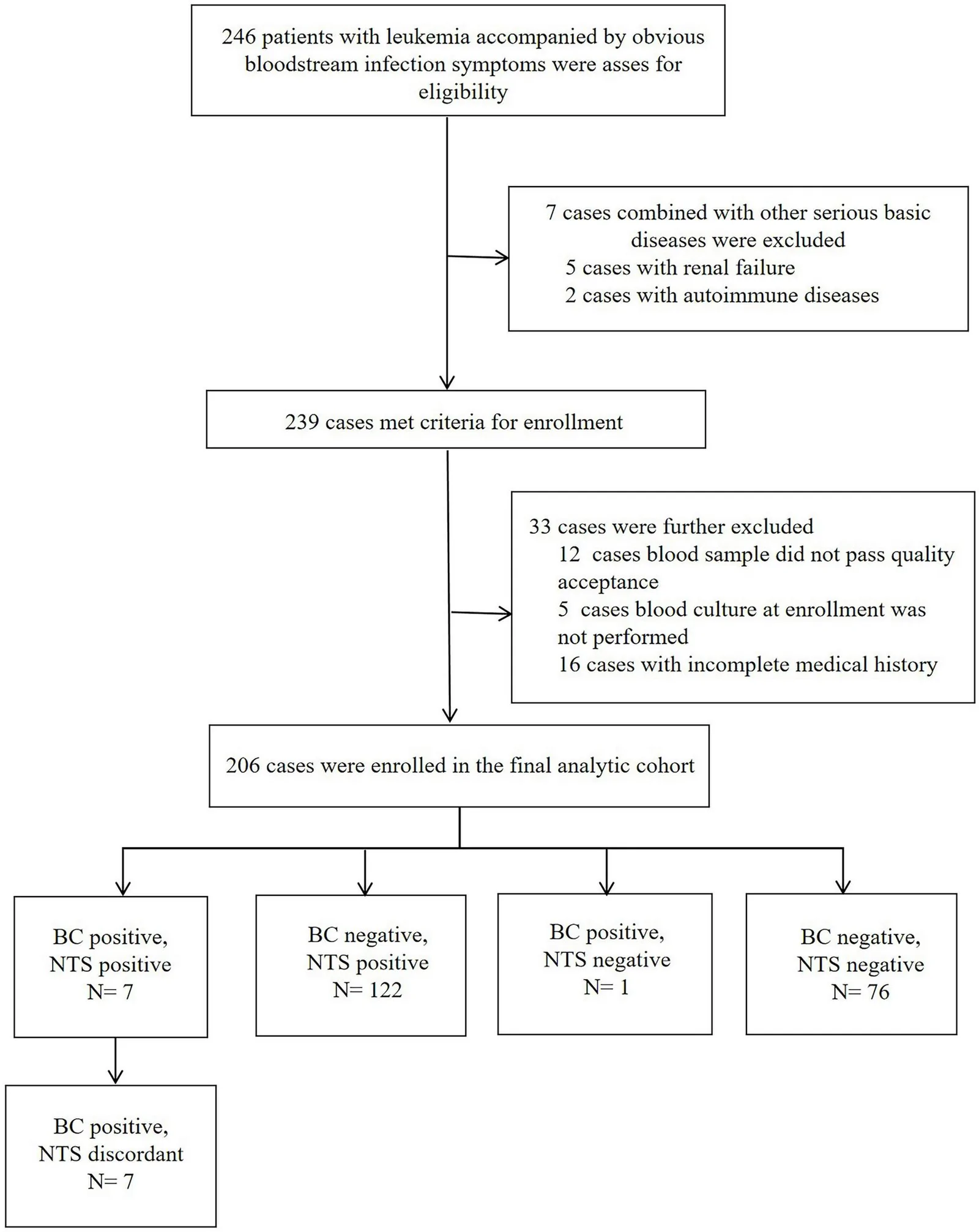
Sankey diagram of clinical medication judgment and therapeutic intervention effect of NTS and BC on patients with suspected infection. Cover All/Not cover all, all/not all pathogens detected by BC and NTS were covered by initial empirical antimicrobial treatment (IEAT). Modified by BC/Modified by NTS/Modified by EXP or FC, antimicrobial therapy was modified by blood culture/NTS/empirical medication or focal specimen culture; Not modified, antimicrobial therapy was not modified. BC, blood culture; EXP, experience treatment; NTS, Nanopore-targeted sequencing. NA, not applicable or not evaluated; Improved: the improvement of clinician-documented improvement made by independent clinicians for each patient based on symptoms and signs, radiological testing results, infection-related laboratory tests, and results of microbiological tests; Not improved: unimproved clinical results recorded by the independent clinician for each patient according to the symptoms and signs, radiological testing results, infection-related laboratory tests, and results of microbiological tests. The hospitalized patients choose to use or not use the initial empirical antimicrobial treatment (IEAT) according to the progress of the disease. The coverage of IEAT on the pathogens of infection was determined according to the results of BC and NTS detection. On this basis, the clinician will adjust the use of antibiotics according to the patient’s vital signs, improvement of symptoms and other conventional laboratory test results. The clinical outcomes of the patients were determined according to the discharge conditions. Interpretation of the Sankey Diagram for Suspected Infection Clinical Pathway: This Sankey diagram visualizes the clinical pathway of 206 hospitalized patients with bloodstream infection, systematically mapping the associations among initial empirical antimicrobial treatment (IEAT) decisions, pathogen detection results, IEAT pathogen coverage, antimicrobial regimen modifications, and final clinical outcomes. It specifically compared the clinical value of nanopore-targeted sequencing (NTS) and conventional blood culture (BC), where the width of each flow corresponds to the number of patients in the corresponding subgroup. The first column represents initial treatment decisions: 181 patients received IEAT before definitive pathogen confirmation, while 25 patients did not initiate empirical antimicrobial therapy. The second column stratifies patients by parallel NTS and BC detection: The largest subgroup (*n* = 122) tested NTS—positive but BC—negative. Additionally, both detection methods yielded negative results for 76 patients, while both yielded positive results for 7 patients. Only 1 patient was BC—positive but NTS—negative. The third column evaluates IEAT coverage of detected pathogens: 92 patients were classified as not applicable (NA), including those without IEAT or with negative pathogen detection. 59 patients had all identified pathogens fully covered by IEAT, whereas 55 patients had incomplete coverage. The fourth column displays treatment adjustment strategies: 158 patients had regimens modified via conventional clinical pathways (empirical adjustment or focal specimen culture). 32 had therapy revised based on the NTS results. 16 received no regimen changes. The final column presents independently adjudicated clinical outcomes: 179 patients achieved clinical improvement. Among them, 29 patients had their treatment plans revised based on the NTS results. 20 showed no clinical improvement. 7 patients died.

Among 206 patients, based on the detection methods, patients were divided into four groups: NTS-positive (NTS+, *n* = 129), NTS-negative (NTS-, *n* = 77), blood culture-positive (BC+, *n* = 8), and blood culture-negative (BC-, *n* = 198). The clinical characteristics of each group are summarized in Supplementary Table S1. In the NTS+ and NTS- groups, the proportions of patients receiving chemotherapy were 93.02% (120/129) and 79.22% (61/77), respectively; while in the BC+ and BC- groups, the proportions were 87.5% (7/8) and 87.88% (174/198), respectively. Regarding immunosuppressant use, the proportions in the NTS+, NTS-, BC+, and BC- groups were 31.01, 31.17, 25.00, and 31.31%, respectively, with no significant differences observed among the groups. On the day of sample collection, the proportion of patients with a body temperature ≥ 39°C was highest in the NTS- group (38.96%, 30/77), which was higher than that in the NTS+ group (27.13%, 35/129). In the BC+ group, 25.00% (2/8) had a temperature ≥ 39°C, compared with 32.32% (64/198) in the BC- group. Regarding CRP levels, the proportion of patients with CRP ≥ 200 mg/L was 13.95% (18/129) in the NTS+ group and 14.29% (11/77) in the NTS- group; in the BC+ group, 37.50% (3/8) had CRP ≥ 200 mg/L, which was significantly higher than that in the BC- group (13.13%, 26/198), half of BC+ cases had CRP 100–200 mg/L. 12.5% BC-positive patients had CRP < 100 mg/L, whereas nearly 60% of NTS-positive patients had CRP < 100 mg/L (Supplementary Table S1). For procalcitonin (PCT) levels > 0.5 ng/mL, the proportions were 19.38% (25/129) in the NTS+ group and 29.87% (23/77) in the NTS- group; in the BC+ group, 62.50% (5/8) had PCT > 0.5 ng/mL, markedly higher than that in the BC- group (21.72%, 43/198). Severe neutropenia (neutrophil count <0.5 × 10^9^/L) was observed in 56.59% (73/129) of the NTS+ group and 44.16% (34/77) of the NTS- group. Notably, all 8 patients (100%) in the BC+ group presented with severe neutropenia, compared with 50% (99/198) in the BC- group.

In summary, NTS-positive and NTS-negative patients showed similar distributions in terms of chemotherapy, immunosuppressant use, and most infection-related indicators. Patients with positive blood cultures (BC+) exhibited more pronounced inflammatory responses (elevated CRP and PCT) and a higher proportion of severe neutropenia.

### 3.2 Performance of NTS relative to BC

The performance of NTS relative to BC was shown in Table 3. Once clinical samples arrive at the laboratory, the turnaround time of NTS detection is less than 19 h (mainly including extraction, library preparation, sequencing, bioinformatic analysis, and report generation. The duration of each procedure is detailed in Supplementary material 2). The electronic report of NTS results was generally sent to the clinician within 24 h. Bacterial or fungal pathogens were identified in 130 participants [63.11% (130/206)], of which 8 [3.88% (8/206)] were BC positive and 129 [62.62% (129/206)] were NTS positive (Figure 2). Among the 130 participants who tested positive for pathogens, 5.38% (7/130) were simultaneously detected by both BC and NTS. Among these 7 participants, 2 were male, with a median age of 58 years (25–65 years) and a median hospital stay of 18 days (10–32 days). The antibiotic treatments administered are shown in Table 4. However, the pathogenic bacteria detected by BC and NTS were not completely consistent, which may be attributed to the difference in detection limits between blood culture and NTS. 0.77% (1/130) were only detected by BC, and 93.85% (122/130) were only detected by NTS. One subject’s NTS false negative refers to patient-level complete NTS negativity versus positive blood culture; separate intrasample organism-level missed pathogens exist in other patients whose overall NTS test remained positive. For patients with prior antibacterial treatment, the detection rate of potential pathogens detected by NTS was 62.36%. For patients without PAT, the detection rate of NTS reached 65.22%. Compared with BC, the NPA of NTS was 38.38% (95%CI: 31.52–45.60%), (76 out of 198 BC negative participants) (Table 3). The BC positivity rate of patients who used antibiotics within 24 h before blood collection was lower than that of those who did not use antibiotics [0.56% (1/180) vs. 21.74% (5/23), *P* < 0.001]. While the NTS positivity rate of patients who used antibiotics within 24 h before blood collection has no significant difference from that of those who did not use antibiotics [61.67% (111/180) vs. 65.22% (15/23), *P* = 0.423].

**TABLE 3:** Contingency between NTS assay and initial BC.

		BC							
		Total (*n* = 206)		No PAT (*n* = 23)		PAT within 24 h (*n* = 180)		PAT over 24 h (*n* = 3)	
Characteristic		Pos	Neg	Pos	Neg	Pos	Neg	Pos	Neg
NTS	Pos	7	122	4	11	1	110	2	1
	Neg	1	76	1	7	0	69	0	0
	PPA	87.50% (47.35–99.68%)		80% (34.26–99.05%)		100% (69.17–100.00%)		–	
	NPA	38.38% (31.52–45.60%)		38.89% (18.31–63.94%)		38.55% (31.30–46.21%)		–	

BC, Blood culture; NTS, Nanopore-targeted sequencing; Pos, Positive; Neg, Negative; PPA, Positive percentage agreement; NPA, Negative percentage agreement; PAT, Prior antibacterial treatment: prior exposure to antibacterial drugs such as imipenem, cilastatin sodium, cefotiam hydrochloride, vancomycin hydrochloride, linezolid, moxifloxacin hydrochloride, cefathiamidine, etc.; CI, Confidence interval.

**TABLE 4:** Positive results of cases detected by both BC and NTS.

Case	BC	NTS	Age	Sex	Antibiotic	Duration of hospital stay (days)	Outcome
1	*Klebsiella oxytoca*	*Staphylococcus aureus, Streptococcus pneumoniae, Escherichia coli, Candida parapsilosis*	25	Male	Imipenem and Cilastatin Sodium, Vancomycin, Voriconazole	10	Improved
2	*Escherichia coli, Enterobacter cloacae*	*Escherichia coli*	35	Female	Imipenem and Cilastatin Sodium, Teicoplanin, Voriconazole	16	Improved
3	*Escherichia coli*	*Haemophilus parainfluenzae*	65	Male	Imipenem and Cilastatin Sodium, Vancomycin, Voriconazole	32	Improved
	*Escherichia coli*	*Acinetobacter johnsonii*	48	Female	Imipenem and Cilastatin Sodium, Vancomycin	18	Improved
5	*Staphylococcus epidermidis*	*Chryseobacterium hominis*	60	Female	Imipenem and Cilastatin Sodium, Vancomycin	28	Improved
6	*Escherichia coli*	*Restrictive malassezia*	59	Female	Meropenem, Voriconazole	27	Improved
7	*Candida tropicalis*	*Radiation-resistant acinetobacter*	58	Female	Imipenem and Cilastatin Sodium, Micafungin	16	Improved

BC, Blood culture; NTS, Nanopore-targeted sequencing.

**FIGURE 2:**
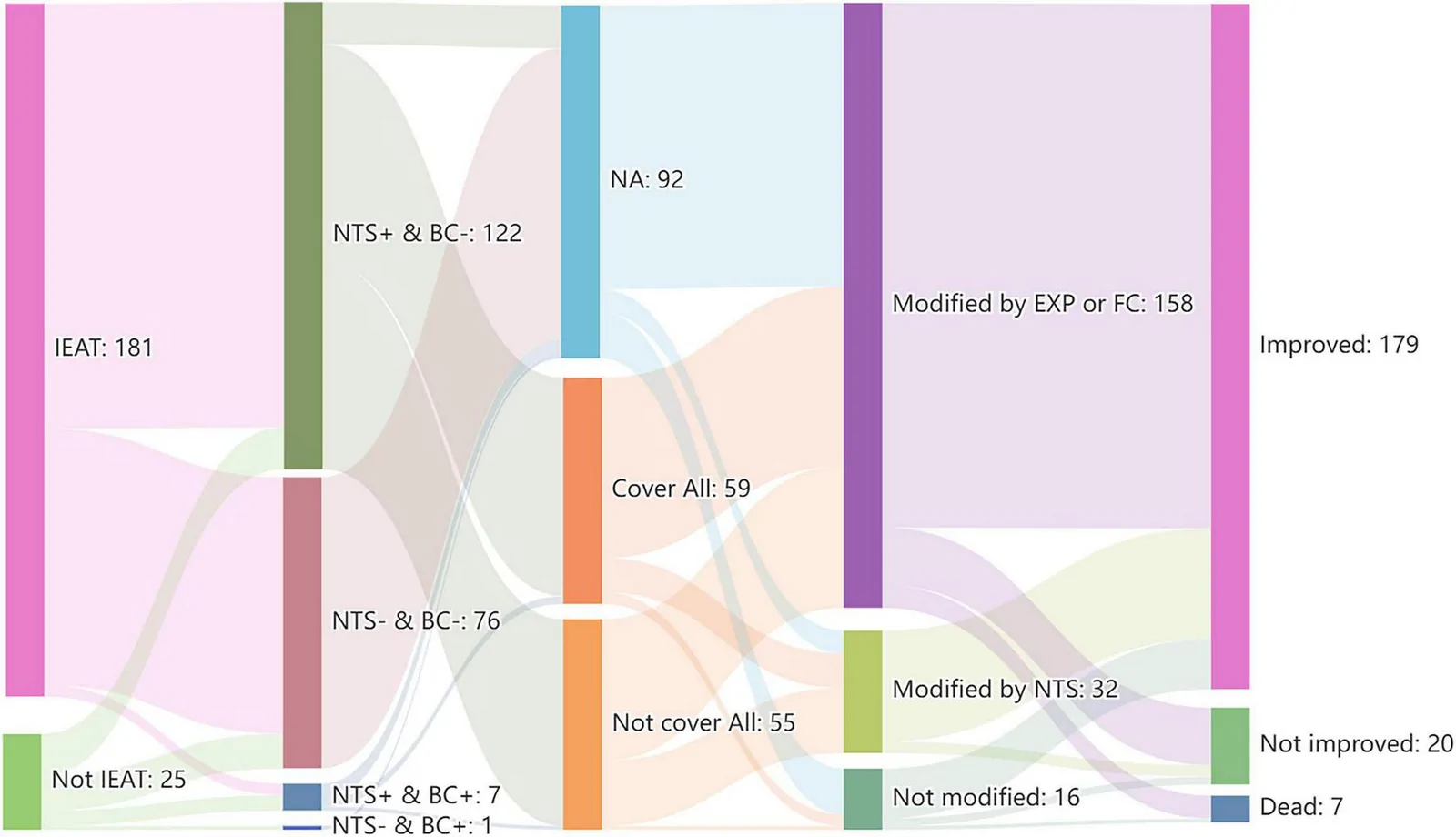
Flowchart for enrollment. Patient enrolment and overall results. BC, Blood culture; NTS, Nanopore-targeted sequencing.

Among the NTS-positive samples, 63.57% (82/129) contained single-pathogen, and 36.43% (47/129) contained multiple-pathogen, compared to 87.50% (95%CI: 47.35–99.68%), (7/8) contained single-pathogen, and only 12.5% (1/8) contained multiple-pathogen detected through conventional blood culture methods.

As shown in Table 5, the sensitivity of NTS for diagnosing bloodstream infections was 65.32% (95%CI: 56.21–73.69%), (81/124), which was significantly higher than BC [6.45% (95%CI: 2.84–12.13%), (8/124)], *P* < 0.001. The specificity of NTS was 41.46% (30.17–53.52%%) (34/82), while that of BC was [100% (95%CI: 95.56–100.00%), (82/82)].

**TABLE 5:** The potential pathogen detection rate of NTS, culture in different types of bloodstream infections.

Characteristic	Bloodstream infection (*n* = 124)	Non-bloodstream infection (*n* = 82)	SN (95%CI)	SP (95%CI)	PPV (95%CI)	NPV (95%CI)
NTS positive	81	48	65.32% (56.21% -73.69%)	41.46% (30.17–53.52%)	62.79% (53.82–71.07%)	44.16% (32.80–56.12%)
NTS negative	43	34				
BC positive	8	0	6.45% (2.84–12.13%)	100.00% (95.56–100.00%)	100.00% (69.17–100.00%)	41.41% (34.40–48.70%)
BC negative	116	82				
*P*			< 0.001	-	0.022	0.572

NTS, Nanopore-targeted sequencing; BC, Blood culture; SN, sensitivity; SP, specificity; PPV, positive predictive value; NPV, negative predictive value. The diagnostic coincidence rate means the consistency between the diagnosis based on the test results of a method (NTS) and the final diagnosis results (based on the typical clinical symptoms of bloodstream infections and microbial culture, etc.).

In general, a total of 64 bacteria and 15 fungi were detected in BC or NTS, of which 64 bacteria and 14 fungi and others were detected in NTS, while only 5 bacteria and 1 fungus were detected in BC. NTS detected an additional 59 types of bacteria and 14 types of fungi in BC negative patients (Table 6). The pathogens detected by NTS and BC of each patient were systematically compared. It was found that 7 patients had inconsistent or incompletely consistent pathogens detected by NTS and BC (Table 4). The pathogenic bacteria identified by BC were *Escherichia coli* (3 cases), *Klebsiella oxytoca* (1 case), *Klebsiella pneumoniae* (1 case), *Enterobacter cloacae* (1 case), *Staphylococcus epidermidis* (1 case), and *Candida tropicalis* (1 case). However, in patients with positive BC results, NTS identified *Staphylococcus aureus* (1 case)*, Streptococcus pneumoniae* (1 case)*, Candida parapsilosis* (1 case)*, Haemophilus parainfluenzae* (1 case)*, Acinetobacter johnsonii* (1 case)*, Chryseobacterium hominis* (1 case)*, Malassezia restricta* (1 case)*, and Acinetobacter radioresistens* (1 case), which were considered to be potentially clinically significant pathogens.

**TABLE 6:** Consistency between NTS and BC for all detected pathogens in 206 patients.

Characteristic	only_BC+	BC+ and NTS+	only_NTS+
Bacteria	7	1	166
*Pseudomonas stutzeri*			17
*Escherichia coli*	3	1	13
*Staphylococcus aureus*			11
*Acinetobacter haemolyticus*			10
*Haemophilus parainfluenzae*			9
*Enterobacter cloacae*	1		8
*Pseudomonas aeruginosa*			8
*Acinetobacter johnsonii*			7
*Acinetobacter radioresistens*			4
*Achromobacter xylosoxidans*			3
*Corynebacterium jeikeium*			3
*Elizabethkingia miricola*			3
*Haemophilus influenzae*			3
*Klebsiella oxytoca*	1		3
*Pseudomonas putida*			3
*Staphylococcus epidermidis*	1		3
*Streptococcus pneumoniae*			3
*Acinetobacter baumannii*			2
*Aerococcus viridans*			2
*Burkholderia cepacia*			2
*Chryseobacterium hominis*			2
*Citrobacter freundii*			2
*Enterobacter tabaci*			2
*Klebsiella pneumoniae*	1		2
*Streptococcus sanguinis*			2
*Acinetobacter calcoaceticus*			1
*Acinetobacter guillouiae*			1
*Anaerococcus vaginalis*			1
*Bacillus circulans*			1
*Bacillus megaterium*			1
*Bacillus pumilus*			1
*Bacillus subtilis*			1
*Bacteroides salanitronis*			1
*Bacteroides thetaiotaomicron*			1
*Campylobacter gracilis*			1
*Cardiobacterium hominis*			1
*Citrobacter braakii*			1
*Citrobacter murliniae*			1
*Corynebacterium kroppenstedtii*			1
*Enterobacter mori*			1
*Enterobacter xiangfangensis*			1
*Enterococcus casseliflavus*			1
*Enterococcus faecalis*			1
*Granulicatella adiacens*			1
*Helicobacter pylori*			1
*Klebsiella aerogenes*			1
*Klebsiella variicola*			1
*Moraxella catarrhalis*			1
*Moraxella nonliquefaciens*			1
*Moraxella osloensis*			1
*Morganella morganii*			1
*Pseudomonas alcaligenes*			1
*Pseudomonas mendocina*			1
*Pseudomonas oryzihabitans*			1
*Rhodococcus degradans*			1
*Serratia marcescens*			1
*Staphylococcus equorum*			1
*Staphylococcus hominis*			1
*Stenotrophomonas maltophilia*			1
*Streptococcus agalactiae*			1
*Streptococcus gordonii*			1
*Streptococcus mitis*			1
*Streptococcus oralis*			1
*Veillonella dispar*			1
Fungi and others	1	0	20
*Alternaria alternata*			3
*Aspergillus penicillioides*			3
*Candida parapsilosis*			2
*Cryptococcus curvatus*			2
*Aspergillus sydowii*			1
*Candida albicans*			1
*Candida krusei*			1
*Candida metapsilosis*			1
*Malassezia globosa*			1
*Malassezia restricta*			1
*Mycoplasma hominis*			1
*Penicillium citrinum*			1
*Trichoderma longibrachiatum*			1
*Yarrowia lipolytica*			1
*Candida tropicalis*	1		0

+, Positive; BC, Blood culture; NTS, Nanopore-targeted sequencing.

The patient-level positivity rate is defined as the proportion of patients who tested positive by either blood culture or NTS, respectively. This metric assesses the clinical sensitivity of each detection method in identifying patients with bloodstream infection (BSI) at the individual level. Among the enrolled patients, 8/206 patients were positive by blood culture, and 129/206 patients were positive by NTS. The patient-level positivity rates were 3.88 and 62.62%, respectively. The proportion of blood culture-confirmed infection cases in which NTS correctly identifies the same pathogen is defined as the organism-level concordance. Among the 8 blood culture-positive specimens, only 1 showed concordant results with NTS, yielding an organism-level concordance rate of 12.5% (Supplementary Table S3)

Among the 122 NTS-positive and BC negative cases, the top five pathogens detected by NTS were *Pseudomonas stutzeri* (17 cases), *Escherichia coli* (13 cases), *Staphylococcus aureus* (11 cases), *Acinetobacter haemolyticus* (11 cases), and *Haemophilus parainfluenzae* (9 cases), respectively. The consistency between the distribution and BC detection results was relatively low. In terms of fungal infection, compared with one case of infection (*Candida tropicalis*) detected by BC, NTS detected 13 pathogens, of which the most common pathogens were *Alternaria alternata* (3 cases) and *Aspergillus penicillioides* (3 cases) (Table 6). *Pseudomonas stutzeri* and rare *Acinetobacter* spp.: These environmental non-fermenters cause catheter-associated low-load bacteremia in neutropenic leukemia patients. Prior antibiotics and competitive overgrowth in culture bottles lead to false-negative BC; patients showed fever resolution after regimen adjustment targeting these species. *Malassezia restricta:* Fastidious lipophilic fungus that fails to grow in standard BC bottles. All positive patients received lipid parenteral nutrition via central venous catheters; fever resolved after lipid suspension and anti-*Malassezia antifungals*. *Alternaria alternata/Aspergillus penicillioides*: Slow-growing environmental molds undetectable by routine blood fungal culture. Patients presented refractory fever with pulmonary invasive mold imaging; identical fungi were recovered from respiratory specimens, and symptoms improved after mold-active antifungal therapy, confirming hematogenous dissemination rather than transient environmental contamination.

### 3.3 Antibiotic treatment and adjustment

Empirical antibiotic treatment before blood collection was administered in the majority of episodes, with rates of 88.37% (NTS+), 89.61% (NTS–), 37.50% (BC+), and 90.91% (BC–). Notably, patients with positive blood culture had a much lower rate of pre-bleeding empirical therapy (37.50%) compared to other groups. Clinical diagnosis of bloodstream infection (BSI) was confirmed in all BC+ episodes 100.00% (95%CI: 69.17–100.00%), whereas BSI was diagnosed in 62.79% (95%CI: 53.82–71.07%) of NTS+ episodes, 55.84% of NTS- episodes, and 58.59% of BC- episodes. Regarding PAT duration, the majority of patients received PAT within 24 h across all groups: 86.05% (NTS+), 89.61% (NTS–), 12.50% (BC+), and 90.40% (BC–). PAT over 24 h was observed in 2.33% of NTS+ episodes and 25.00% of BC+ episodes, but none in the NTS- group. Positive blood culture results serve as the microbiological comparator for diagnosing bloodstream infections. In cases where blood culture yields a positive result, antibiotics are modified based on the drug susceptibility profile of the isolated pathogen. Conversely, when blood culture is negative while NTS returns a positive result, clinicians make a comprehensive assessment by evaluating the patient’s clinical manifestations of bloodstream infection alongside laboratory infection markers, in order to ascertain whether the NTS-detected microorganism represents the true pathogenic agent, and given that the NTS assay does not include antimicrobial susceptibility testing, antibiotic adjustments are made based on the pathogen detection results and clinical experience. NTS results prompted antibiotic adjustments in a substantial proportion of NTS-positive episodes (14.08%), whereas blood culture results had a limited role in guiding timely adjustments, likely due to its low positivity rate (8/206, 3.9%) and longer turnaround time. Clinical experience remained the predominant driver of antibiotic modification across all four groups, even when the empirical regimen already covered the identified pathogens (Supplementary Table S2).

### 3.4 Clinical effects of NTS

We compared the results of NTS and BC with the final clinical diagnosis. It was found that the consistency between NTS results and the final clinical diagnosis was 55.83% compared to the BC result of 43.69%.

Of all NTS or BC positive cases, the IEAT of 59 cases [45.74% (59 of 129)] covered the indicated pathogens. The 55 cases [42.64% (55 of 129)] of IEAT did not cover or only covered a part of pathogens detected by NTS. The medication of 32 participants [24.81% (32/129)] was modified by NTS results (Figure 2). Among the 206 cases, 32 cases adjusted for medication based on NTS results, and 29 participants showed the bloodstream infection improved clinical results, while the status of bloodstream infection of 3 participants (2.33%) was not effectively brought under control.

## 4 Discussion

NTS detection technology has gained significant attention in the diagnosis of secondary bloodstream infections associated with hematologic diseases (Hong et al., 2023; Long et al., 2025). Traditional blood culture methods demonstrated low positivity rates for pathogenic bacteria in leukemia bloodstream infections, with some studies reporting rates below 20% (Bissonnette and Bergeron, 2012; Mandell et al., 2007; Waterer and Wunderink, 2001). NTS delivered higher pathogen detection positivity and sensitivity than conventional blood culture. However, increased detection positivity does not necessarily translate to improved diagnostic accuracy. As a high-sensitivity auxiliary detection tool, NTS can assist in screening potential pathogens and facilitating individualized antimicrobial selection, while its diagnostic validity requires comprehensive clinical validation. These observations tend to underscore that reliable pathogen detection approaches are likely to carry substantial value within contemporary clinical care.

In our study, NTS detected a range of pathogens, including those commonly and infrequently reported bloodstream infections. This indicates that NTS has a wider detection spectrum and can identify more pathogens in adult bloodstream infection cases. Overall, NTS detected an additional 59 types of bacteria and 14 types of fungi in BC negative patients. Notably, such incremental positive detections only reflect superior detection sensitivity, rather than superior diagnostic accuracy, and these newly identified microbes need clinical assessment to rule out non-pathogenic colonization or contamination.

For patients with prior antibacterial treatment, the detection rate of potential pathogens detected by NTS was significantly higher than that of BC, with a detection rate of 62.36%. For patients without PAT, the detection rate of NTS reached 65.22%, which was consistent with previous studies (Han et al., 2024; Hong et al., 2023; Zhao et al., 2025). NTS shows certain advantages in improving the positive detection rate of pathogens in suspected bloodstream infection cases, particularly in antibiotic-exposed patients. Nevertheless, the enhanced detection rate and positivity do not equate to improved diagnostic accuracy. NTS compensates for the false-negative limitations of traditional culture caused by empirical antibiotic therapy, yet all positive findings require clinical differentiation to ensure diagnostic reliability.

The study further verified the favorable detection performance of NTS for single-pathogen and multiple-pathogen infections. Among the NTS-positive samples, 36.43% contained multiple pathogens, compared to only 12.5% that contained multiple pathogens detected through conventional blood culture methods, which was consistent with previous findings (Jiang et al., 2023; Luo et al., 2025). The bloodstream infection cohort with multiple invasive devices and immune compromise is clinically predisposed to mixed bloodstream infections, which may contextualize the high proportion. These results indicate that NTS is sensitive in detecting mixed pathogens and can identify more co-infecting pathogens missed by traditional culture. BC only identifies high-burden, viable monomicrobial bacteremia, missing mixed, low-grade, or fastidious infections common in immunocompromised hosts, while NTS frequently identified multiple co-circulating bacteria and fungi. Blood culture is constrained by selective growth media, incubation conditions, and competitive overgrowth of fast-growing species that outcompete fastidious, slow-growing, or fungal pathogens. NTS, by contrast, agnostically amplifies all microbial nucleic acid in the blood sample, unveiling mixed pathogen communities missed by culture. But at the same time, the clinical significance of multiple detected pathogens should be carefully judged by clinicians.

In this study, among the 206 cases, 32 cases adjusted for medication based on NTS results, and 29 participants showed the bloodstream infection improved clinical results. This suggests the significance of early implementation of NTS detection in leukemia patients with bloodstream infection. However, the observational nature of the study and the absence of a comparator group preclude attributing these improvements directly to treatment guided by different detection methods. Empirical broad-spectrum antimicrobial therapy may promote the emergence of drug-resistant bacteria (Duan et al., 2016), complicating diagnosis while increasing the prevalence of resistant strains and imposing greater financial burdens on patients. Therefore, NTS can serve as a valuable auxiliary detection tool to compensate for the underdetection of traditional blood culture.

All BC-positive patients (100%) presented with neutropenia < 0.5 × 10^9^/L, whereas only 56.59% of NTS-positive patients had severe neutropenia. Severe granulocytopenia impairs pathogen clearance, allowing sustained viable pathogen circulation detectable by BC without antibiotic interference, yet NTS still detected pathogens acid fragments in over 40% of non-severely neutropenic patients, demonstrating NTS’s higher sensitivity in milder neutrophil suppression patients. 62.5% of BC-positive patients had PCT > 0.5 ng/mL vs. only 19.38% of NTS-positive cases. BC positivity strongly correlates with robust systemic bacterial sepsis marked by high PCT, while NTS frequently identifies pathogens in patients with milder subclinical infection with low-to-moderate PCT (0.05–0.5 ng/mL, 70.54% NTS+). 12.5% BC-positive patients had CRP < 100 mg/L, whereas nearly 60% of NTS-positive patients had CRP < 100 mg/L. It is difficult for conventional BC to capture low-grade inflammatory bloodstream pathogens readily detectable by NTS.

Though NTS shows utility in bloodstream infection diagnosis and therapy, it also possesses inherent limitations like non-negligible false-positive results (Bialasiewicz et al., 2019). Greater positivity alone does not guarantee improved diagnostic accuracy, as many detected microbes may merely be contaminants, colonizers or non-pathogenic organisms lacking clinical significance. Clinical decisions on pathogen identification should not rest solely on NTS findings. In addition, NTS only provides pathogen results and fails to substitute blood culture for phenotypic antibiotic susceptibility testing. NTS alone cannot replace the necessity of pathogen detection through methods like culture. Clinicians should not solely rely on NTS results for pathogen identification, and NTS can only predict test results that cannot replace blood culture to obtain phenotypic antimicrobial susceptibility results (Liu et al., 2024). Therefore, clinicians should integrate patient history with complementary diagnostic modalities to accurately identify the causative pathogen, inform the diagnosis, and guide subsequent treatment strategies.

It must be acknowledged that the high sensitivity of NTS makes it highly susceptible to detecting background microorganisms and procedural contaminants. To avoid overestimating the diagnostic value of NTS, we strictly adhered to the principle of “clinical predominance, sequencing as corroboration,” comparing sequence detections with sepsis-related clinical phenotypes (including organ dysfunction, markedly elevated inflammatory markers, positive imaging findings, and pathogen detection in other specimens). However, the pathogenic threshold for a single detection currently lacks a standardized definition, underscoring the need for future studies to establish standardized negative/positive background control databases in order to precisely define the clinical significance thresholds of different microorganisms in immunocompromised populations. The BC positivity rate in this study was only 3.88%, much lower than that of NTS. This low BC positivity is mainly attributed to the widespread prior antibiotic use among neutropenic leukemia patients and suboptimal clinical operation conditions rather than microbiological confirmation. Conventional blood culture is insufficient for pathogen screening in this high-risk population and NTS detection results must be comprehensively evaluated clinically to confirm diagnostic validity. Although NTS achieved good sensitivity for bloodstream infection screening, its low specificity (41.46%) is a major limitation. Many patients without definitive bloodstream infection exhibited positive NTS results. We are unable to quantify the frequency of subsequent unnecessary antimicrobial escalation, extra testing, increased costs and drug toxicity due to insufficient follow-up data in this retrospective study. Nevertheless, false-positive NTS results bear clear clinical risks: premature escalation of antimicrobials may lead to toxicity, dysbiosis and antimicrobial resistance, while additional confirmatory tests increase medical expenses. The high rate of false positives among leukemia patients could be explained by microbial translocation and colonization. Therefore, NTS results require multidisciplinary comprehensive interpretation rather than isolated use for therapeutic decision-making. NTS-informed antimicrobial adjustments and subsequent therapeutic improvement were both factored into clinical infection adjudication, creating a circular logic that may overestimate the assay’s diagnostic accuracy and its clinical impact and we need to avoid this confusion in the future independent verification queue.

Our research has some limitations. First of all, we did not investigate or conclude the detection of a suspicious bloodstream infection caused by a virus in our study. Secondly, we use preliminary data for analysis, but still lack health control. In future research, we will further explore the detection of viral infection in patients with bloodstream disease by NTS. At the same time, there is a lack of guidance on clinical drug selection for the ability of NTS to detect drug-resistant genes in infectious pathogens. In addition, since the results of NTS may be easily affected by many factors, the standards in our retrospective, single-center diagnostic observational study should be thoroughly revised and tested before being applied to other centers. And access to NTS results during clinical diagnosis adjudication may inflate the calculated sensitivity, specificity and perceived clinical utility of the assay.

## 5 Conclusion

In conclusion, this retrospective, single-center diagnostic observational study of leukemia patients with suspected bloodstream infection demonstrates that NTS targeting bacterial 16S rRNA and fungal ITS regions can improve the detection yield of bacterial and fungal pathogens compared with conventional blood culture, particularly among patients who received prior empirical antimicrobial therapy. However, the assay exhibits low specificity, accompanied by incomplete contamination controls, uncertain positivity thresholds. These key limitations preclude the overall diagnostic advantages of NTS as an independent detection method. NTS may provide useful adjunctive information on bacterial and fungal pathogens, but NTS can only serve as a supplementary diagnostic tool, and all sequencing results must be comprehensively adjudicated alongside clinical manifestations, routine blood culture data, stringent background contamination assessment, and antimicrobial susceptibility testing based on blood culture before guiding therapeutic decisions. Future multicenter prospective investigations are required to establish unified standardized operating criteria and clinical interpretive guidelines for NTS testing in leukemia patients at risk of bloodstream infection.

## Data Availability

The data analyzed in this study is subject to the following licenses/restrictions: If you need the original dataset, please contact the corresponding author. Requests to access these datasets should be directed to morgan@whu.edu.cn.

## Footnotes

1
https://www.lddgo.net/chart/sankey-chart

## Supplementary material

Supplementary Figure S1 Flowchart of the filtering step for laboratory contamination. Three negative controls were designed for filtering out bacteria and fungi contaminants from NTS laboratory sampling and from human normal flora named Long-term NC, Dynamic NC, and Batch NC. Organisms were kept only if they were found > LNC-FC/DNC/BNC in samples than in controls. Then a list of organisms (called the PCR-organism list) was used to filter out contaminants introduced by PCR.“Long-termNC”(DNA extraction control, PCR Negative control under surveillance for 10 months, and samples from healthy individuals), “DynamicNC” (negative controls within 30 days were collected according to the sequencing date of each experimental sample and samples from healthy individuals), and “BatchNC” (negative controls batched in same sequencing library). The ratio of read abundance in a sample to read abundance in the BatchNC/DynamicNC/Long-termNC used to determine whether an organism was a contaminant from the laboratory, was named as “Long-termNC-FoldChange”(LNC-FC), “DynamicNC-FoldChange” (DNC-FC) and “BatchNC-FoldChange” (BNC-FC), respectively.

## References

[B1] Bialasiewicz S. Duarte T. P. S. Nguyen S. H. Sukumaran V. Stewart A. Appleton S. et al. (2019). Rapid diagnosis of *Capnocytophaga canimorsus* septic shock in an immunocompetent individual using real-time Nanopore sequencing: a case report. *BMC Infect. Dis.* 19:660. 10.1186/s12879-019-4173-2 31340776 PMC6657077

[B2] Bissonnette L. Bergeron M. G. (2012). Infectious disease management through point-of-care personalized medicine molecular diagnostic technologies. *J. Pers. Med.* 2, 50–70. 10.3390/jpm2020050 25562799 PMC4251365

[B3] Blauwkamp T. A. Thair S. Rosen M. J. Blair L. Lindner M. S. Vilfan I. D. et al. (2019). Analytical and clinical validation of a microbial cell-free DNA sequencing test for infectious disease. *Nat. Microbiol.* 4, 663–674. 10.1038/s41564-018-0349-6 30742071

[B4] Carandang T. H. D. C. Cunanan D. J. Co G. S. Pilapil J. D. Garcia J. I. Restrepo B. I. et al. (2025). Diagnostic accuracy of nanopore sequencing for detecting Mycobacterium tuberculosis and drug-resistant strains: a systematic review and meta-analysis. *Sci. Rep.* 15:11626. 10.1038/s41598-025-90089-x 40185766 PMC11971303

[B5] Chamilos G. Lionakis M. S. Kontoyiannis D. P. (2018). Call for action: invasive fungal infections associated with ibrutinib and other small molecule kinase inhibitors targeting immune signaling pathways. *Clin. Infect. Dis.* 66, 140–148. 10.1093/cid/cix687 29029010 PMC5850040

[B6] Chen Y. F. Li J. Xu L. L. Gãman M. A. Zou Z. Y. (2023). Allogeneic stem cell transplantation in the treatment of acute myeloid leukemia: An overview of obstacles and opportunities. *World J. Clin. Cases* 11, 268–291. 10.12998/wjcc.v11.i2.268 36686358 PMC9850970

[B7] Chinese Society of Hematology, Chinese Medical Association, and Chinese Medical Doctor Association, Hematology Branch (2016). Chinese guidelines for the clinical application of antibacterial drugs for agranulocytosis with fever (2016). *Zhonghua Xue Ye Xue Za Zhi* 37:353e9. 10.3760/cma.j.issn.0253-2727.2016.05.001 27210867 PMC7348317

[B8] Cunanan D. J. Carandang T. H. D. C. Pilapil J. D. Cunanan D. J. Mollasgo A. G. Manalo G. N. S. et al. (2026). Nanopore sequencing for microbiological diagnosis of bacterial pneumonia: A systematic review and meta-analysis. *Eur. J. Clin. Microbiol. Infect. Dis.* 45, 1077–1091. 10.1007/s10096-025-05387-z 41499025

[B9] Duan J. Xie Y. Yang J. Luo Y. Guo Y. Wang C. (2016). Variation of circulating inflammatory mediators in *Staphylococcus aureus* and *Escherichia coli* bloodstream infection. *Med. Sci. Monit.* 22, 161–171. 10.12659/msm.896576 26772168 PMC4720174

[B10] Florio W. Morici P. Ghelardi E. Barnini S. Lupetti A. (2018). Recent advances in the microbiological diagnosis of bloodstream infections. *Crit. Rev. Microbiol.* 44, 351–370. 10.1080/1040841X.2017.1407745 29185372

[B11] Freifeld A. G. Bow E. J. Sepkowitz K. A. Boeckh M. J. Ito J. I. Mullen C. A. et al. (2011). Clinical practice guideline for the use of antimicrobial agents in neutropenic patients with cancer: 2010 update by the Infectious Diseases Society of America. *Clin. Infect. Dis.* 52, e56–e93. 10.1093/cid/cir073 21258094

[B12] Gu W. Deng X. Lee M. Sucu Y. D. Arevalo S. Stryke D. et al. (2021). Rapid pathogen detection by metagenomic next-generation sequencing of infected body fluids. *Nat. Med.* 27, 115–124. 10.1038/s41591-020-1105-z 33169017 PMC9020267

[B13] Han D. Yu F. Zhang D. Hu J. Zhang X. Xiang D. et al. (2024). Molecular rapid diagnostic testing for bloodstream infections: Nanopore targeted sequencing with pathogen-specific primers. *J. Infect.* 88:106166. 10.1016/j.jinf.2024.106166 38670268

[B14] Hong M. Peng D. Fu A. Wang X. Zheng Y. Xia L. et al. (2023). The application of nanopore targeted sequencing in the diagnosis and antimicrobial treatment guidance of bloodstream infection of febrile neutropenia patients with hematologic disease. *J. Cell Mol. Med.* 27, 506–514. 10.1111/jcmm.17651 36722317 PMC9930421

[B15] Jiang S. Wei Y. Ke H. Song C. Liao W. Meng L. et al. (2023). Building a nomogram plot based on the nanopore targeted sequencing for predicting urinary tract pathogens and differentiating from colonizing bacteria. *Front. Cell Infect. Microbiol.* 13:1142426. 10.3389/fcimb.2023.1142426 37265501 PMC10229875

[B16] Jing C. Chen H. Liang Y. Zhong Y. Wang Q. Li L. et al. (2021). Clinical evaluation of an improved metagenomic next-generation sequencing test for the diagnosis of bloodstream infections. *Clin. Chem.* 67, 1133–1143. 10.1093/clinchem/hvab061 34060627

[B17] Liu P. Y. Wu H. C. Li Y. L. Cheng H. W. Liou C. H. Chen F. J. et al. (2024). Comprehensive pathogen identification and antimicrobial resistance prediction from positive blood cultures using nanopore sequencing technology. *Genome Med.* 16:141. 10.1186/s13073-024-01416-2 39617907 PMC11610257

[B18] Long J. Zeng B. Li J. Zhang J. Deng G. (2025). Global trends in the application of nanopore sequencing technology in the detection of infectious disease pathogens: a bibliometric analysis from 2014 to 2024. *Front. Med.* 12:1610063. 10.3389/fmed.2025.1610063 40771470 PMC12325371

[B19] Luo Y. Cheng W. Ma L. Wang T. Shi W. (2025). Diagnostic value of nanopore-based metagenomic third-generation sequencing in the diagnosis of *Pneumocystis jirovecii* infection in patients with lung cancer. *J. Med. Microbiol.* 74:002031. 10.1099/jmm.0.002031 40607500 PMC12224071

[B20] Mandell L. A. Wunderink R. G. Anzueto A. Bartlett J. G. Campbell G. D. Dean N. C. et al. (2007). Infectious diseases society of America/American thoracic society consensus guidelines on the management of community-acquired pneumonia in adults. *Clin. Infect. Dis.* 44(Suppl. 2), S27–S72. 10.1086/511159 17278083 PMC7107997

[B21] Marchesi F. Pimpinelli F. Ensoli F. Mengarelli A. (2018). Cytomegalovirus infection in hematologic malignancy settings other than the allogeneic transplant. *Hematol. Oncol.* 36, 381–391. 10.1002/hon.2453 28660653

[B22] Ministry of Health of the People’s Republic of China, (2001). Diagnostic criteria for hospital infection (Trial). *Natl. Med. J. China* 81, 314–320. Available online at: https://d.wanfangdata.com.cn/periodical/CiBQZXJpb2RpY2FsQ0hJU29scjkyMDI2MDkxMzIwNDYxNxINemh5eDIwMDEwNTAyNxoIcG5yN2JyMXU

[B23] Rapszky G. A. Do To U. N. Kiss V. E. Kói T. Walter A. Gergõ D. et al. (2025). Rapid molecular assays versus blood culture for bloodstream infections: a systematic review and meta-analysis. *EClinicalMedicine* 79:103028. 10.1016/j.eclinm.2024.103028 39968206 PMC11833021

[B24] Rhodes A. Evans L. E. Alhazzani W. Levy M. M. Antonelli M. Ferrer R. et al. (2017). Surviving sepsis campaign: international guidelines for management of sepsis and septic shock: 2016. *Intens. Care Med.* 43, 304–377. 10.1007/s00134-017-4683-6 28101605

[B25] Sheka D. Alabi N. Gordon P. M. K. (2021). Oxford nanopore sequencing in clinical microbiology and infection diagnostics. *Brief Bioinform.* 22:bbaa403. 10.1093/bib/bbaa403 33483726

[B26] Society of Critical Care Medicine and Chinese Medical Association (2007). Guidelines for hemodynamic monitoring and support of severe infection and septic shock in Adults (2006). *Chin. J. Emerg. Med.* 16, 121–126. 10.3760/j.issn:1671-0282.2007.02.003

[B27] Society of Critical Care Medicine, and Chinese Medical Association (2015). Chinese guidelines for the treatment of severe sepsis/septic shock (2014). *Chin. Crit. Care Med.* 2015, 401–426. Available online at: https://d.wanfangdata.com.cn/periodical/CiBQZXJpb2RpY2FsQ0hJU29scjkyMDI2MDkxMzIwNDYxNxISemd3emJqanl4MjAxNTA2MDAxGghndWJjOTFrNA

[B28] Society of Critical Care Medicine, and Chinese Medical Association (2008). Guidelines for prevention and treatment of intravascular catheter associated infections (2007). *Chin. J. Intern. Med.* 47, 691–699. Available online at: https://d.wanfangdata.com.cn/periodical/CiBQZXJpb2RpY2FsQ0hJU29scjkyMDI2MDkxMzIwNDYxNxINemhuazIwMDgwODAzMRoINm9iYnFwcG0

[B29] Waterer G. W. Wunderink R. G. (2001). The influence of the severity of community-acquired pneumonia on the usefulness of blood cultures. *Respir. Med.* 95, 78–82. 10.1053/rmed.2000.0977 11207022

[B30] Zhao M. Ouyang Y. Mei J. Liu H. (2025). Enhancing pathogens detection in suspected geriatric bloodstream infections using Nanopore-targeted sequencing. *Microbiol. Spectr.* 13:e0155424. 10.1128/spectrum.01554-24 39576187 PMC11705817

