# Supplementary material for: Application and evaluation of nanopore targeted sequencing in the detection of bacterial and fungal pathogens in leukemia bloodstream infections
Source: Front Microbiol. 2026 Sep 24;17:1866353. doi: 10.3389/fmicb.2026.1866353 (PMC13682514; doi:10.3389/fmicb.2026.1866353)
Supplement: Supplementary file 1 [file Supplementary_file_1.doc]

**Table S1.** Basic clinical characteristics of NTS negative, NTS positive, BC negative, BC positive patients.

| **Characteristic** | **NTS +（129）** | **NTS - （77）** | **BC +（8）** | **BC -（198）** |
| --- | --- | --- | --- | --- |
| Chemotherapy |  |  |  |  |
| Yes | 120 (93.02%) | 61 (79.22%) | 7 (87.5%) | 174 (87.88%) |
| No | 9 (6.98%) | 16 (20.78%) | 1 (12.5%) | 24 (12.12%) |
| Immunosuppressor |  |  |  |  |
| Yes | 40 (31.01%) | 24 (31.17%) | 2 (25.00%) | 62 (31.31%) |
| No | 89 (69.00%) | 53 (58.83%) | 6 (75.00%) | 136 (68.69%) |
| Infection indicators on the day of samples collection | | | | |
| Temperature (°C) |  |  |  |  |
| ＜38 | 52 (40.31%) | 26 (33.77%) | 5 (62.5%) | 73 (36.87%) |
| 38-39 | 41 (31.78%) | 21 (27.27%) | 1 (12.5%) | 61 (30.81%) |
| ≥39 | 35 (27.13%) | 30 (38.96%) | 2 (25%) | 64 (32.32%) |
| CRP (mg/L) |  |  |  |  |
| ＜100 | 77 (59.69%) | 41 (53.25%) | 1 (12.5%) | 117 (59.09%) |
| 100-200 | 34 (26.36%) | 25 (32.47%) | 4 (50%) | 55 (27.78%) |
| ≥200 | 18 (13.95%) | 11 (14.29%) | 3 (37.5%) | 26 (13.13%) |
| Procalcitonin (PCT, ng/mL) | | | | |
| ≤0.05 | 13 (10.08%) | 6 (7.80%) | 0 (0%) | 19 (9.60%) |
| 0.05-0.5 | 91 (70.54%) | 48 (62.34%) | 3 (37.5%) | 136 (68.69%) |
| ＞0.5 | 25 (19.38%) | 23 (29.87%) | 5 (62.5%) | 43 (21.72%) |
| Neutrophile granulocyte (x10^9^/L) | | | | |
| ＜0.5 | 73 (56.59%) | 34 (44.16%) | 8 (100%) | 99 (50%) |
| ≥0.5 | 56 (43.10%) | 42 (54.55%) | 0 (0%) | 99 (50%) |

Abbreviations: BC, Blood culture; NTS, Nanopore-targeted sequencing; CRP, C-reactive protein.

**Table S2.** Antibiotics use and adjustment in patients with NTS negative, NTS positive, BC negative, BC positive results.

| **Characteristic** | **NTS +（129）** | **NTS - （77）** | **BC +（8）** | **BC -（198）** |
| --- | --- | --- | --- | --- |
| Empirical antibiotic treatment before blood collection | | | | |
| Yes | 114 (88.37%) | 67 (87.01%) | 3 (37.50%) | 178 (89.90%) |
| No | 15 (11.63%) | 10 (12.99%) | 5 (62.50%) | 20 (10.10%) |
| Clinical Diagnosis | | | | |
| Bloodstream infection | 81 (62.79%) | 43 (55.84%) | 8 (100.00%) | 116 (58.59%) |
| Non-Bloodstream infection | 48 (37.21%) | 34 (44.16%) | 0 (0.00%) | 82 (41.41%) |
| Prior antibacterial treatment (PAT) duration | | | | |
| No PAT | 15 (11.63%) | 10 (12.99%) | 5 (62.50%) | 20 (10.10%) |
| PAT within 24 h | 111 (86.05%) | 67 (87.01%) | 1 (12.50%) | 177 (89.39%) |
| PAT before 24 h | 3 (2.33%) | 0 (0.00%) | 2 (25.00%) | 1 (0.51%) |
| Antibiotic Adjustment | | | | |
| Modified by NTS/BC | 32 (24.81%) | 0 (0.00%) | 1 (12.50%) | 31 (15.66%) |
| Not modified (Antibiotic regimen covering pathogens) | 6 (4.65%) | 10 (12.99%) | 0 (0.00%) | 16 (8.08%) |
| Modified by EXP, FC or other infection markers (Antibiotic regimen covering pathogens) | 91 (70.54%) | 67(87.01%) | 7 (87.50%) | 151 (76.26%) |

Abbreviations: BC, Blood culture; NTS, Nanopore-targeted sequencing; Modified by NTS/BC：When empirical antibiotic therapy did not cover or only partially covered pathogens detected by NTS or blood culture, adjustments were made based on test results covered pathogens detected by NTS or blood culture, adjustments were made based on test results; Not modified (Antibiotic regimen covering pathogens)：When empirical therapy covered the pathogens and was clinically effective, no adjustment was made; Modified by EXP, FC or other infection markers (Antibiotic regimen covering pathogens): When empirical therapy covered the pathogens but clinical response was poor, adjustments were made based on clinical experience (EXP), focal specimen culture or other infection markers.

**Table S3.** Positive results of cases detected by both BC and NTS.

| **Analysis Level** | **Metric** | **Blood Culture** | **NTS** | **Agreement / Note** |
| --- | --- | --- | --- | --- |
| Patient-level | Positive cases (n, %) | 8 (3.88%) | 129 (62.62%) | NTS detected more patients |
| Organism-level | Identical species identified (paired samples, n/N, %) | - | - | 1/8 (12.50%) concordance |

**Figure S1.** Flowchart of the filtering step for laboratory contamination.

Three negative controls were designed for filtering out bacteria and fungi contaminants from NTS laboratory sampling and from human normal flora named Long-term NC, Dynamic NC, and Batch NC. Organisms were kept only if they were found >LNC-FC/DNC/BNC in samples than in controls. Then a list of organisms (called the PCR-organism list) was used to filter out contaminants introduced by PCR.“Long-termNC”(DNA extraction control, PCR Negative control under surveillance for 10 months, and samples from healthy individuals), “DynamicNC”(negative controls within 30 days were collected according to the sequencing date of each experimental sample and samples from healthy individuals), and “BatchNC”(negative controls batched in same sequencing library).

The ratio of read abundance in a sample to read abundance in the BatchNC/DynamicNC/Long-termNC used to determine whether an organism was a contaminant from the laboratory, was named as “Long-termNC-FoldChange”(LNC-FC), “DynamicNC-FoldChange”(DNC-FC) and “BatchNC-FoldChange”(BNC-FC), respectively.
