# Supplementary material for: Application and evaluation of nanopore targeted sequencing in the detection of bacterial and fungal pathogens in leukemia bloodstream infections
Source: Front Microbiol. 2026 Sep 24;17:1866353. doi: 10.3389/fmicb.2026.1866353 (PMC13682514; doi:10.3389/fmicb.2026.1866353)
Supplement: Supplementary file 2 [file Supplementary_file_2.doc]

**Methodological Explanation of NTS**

1. **Detailed Specifications of the NTS Platform and Positive Identification Criteria**

**1.1 Target Amplified Regions**

This nanopore targeted sequencing (NTS) platform adopts distinct genetic regions as amplification targets for different types of pathogens. Conserved segments within the 16S rRNA gene are targeted for bacterial detection, conserved regions of the ITS1/ITS2 internal transcribed spacer are selected for fungal identification. All candidate primers are screened from conserved sequences located within 50 base pairs upstream and downstream of classic anchor primer sites.

#### **1.2 Primer Sets**

Classic 27F/1492R and ITS1/ITS4 serve as the core anchor primers for bacterial 16S rRNA and fungal ITS1/2 respectively. Anchor primers are combined with supplementary primers at a molar ratio of 3:1 to form the final universal primer pairs. Strict criteria are applied for primer screening: primer length is controlled between 18 and 30 base pairs, melting temperature ranges from 58 °C to 65 °C with a temperature difference below 3 °C between paired primers, GC content is maintained at 40% to 60%, and the Gibbs free energy of the last five bases at the 3' terminus is ≥ −9 kcal/mol. To enable high-throughput multiplex detection across multiple samples, 96 unique barcode sequences are added to the 5' end of all primers.

**1.3 Pathogen Coverage**

The customized multiplex amplicon panel covers a broad spectrum of common pathogens responsible for clinical infectious diseases. A reportable list of clinical pathogens was set up referred to published papers which apply next generation sequencing to identify the pathogens in sepsis with 209 fungi and 762 bacteria (https://kariusdx.com/karius-test/pathogens). The platform is capable of simultaneous detection of bacteria and fungi. The performance of the panel is further validated using a standard microbial community consisting of eight bacterial and two fungal strains.

**1.4 Reference Database**

All reference databases are curated based on public resources from the National Center for Biotechnology Information (NCBI). Independent databases are constructed for bacterial 16S rDNA, fungal ITS1/2 sequences, and Mycobacterium rpoB gene sequences. Redundant sequences and artificially synthesized sequences are thoroughly removed during database curation to ensure the reliability of reference datasets.

**1.5 Bioinformatic Workflow**

Basecalling and quality assessment of sequencing data were performed using Oxford Nanopore GridION X5 and Guppy in high accuracy mode (ont-guppy-for-gridion v.1.4.3-1 and v.3.0.3-1; high-accuracy basecalling mode). Sequencing reads with undesired length (<200 nt or >2000 nt) were discarded. An in-house script was used to analyze the output of the basecalling data and generate a real-time taxonomy list of each sample by screening and starting the bioinformatic pipeline when every 4000 reads passed the base calling process. Adapter and barcode sequences are trimmed and separated by Porechop (v.0.2.4) to demultiplex samples. The reads of each sample were mapped against the reference database using BLASTn (v.2.9.0+). Reads mapped against the reference database of each sample were filtered by coverage >90%. Then, the taxonomy of each read was assigned according to the taxonomic information of the mapped subject sequence. For the reads preliminary assigned to the same species, a consensus sequence was generated using Medaka (v.0.10.1). Then, the consensus sequence was remapped to the reference database, and the best assigned taxon was used as the final detection result of reads from the same species of the preliminary taxonomy assignment. The taxonomy list generated from each of the 4,000 reads that passed the basecalling process was integrated into the previous taxonomy list, generating an up-to-date taxonomy list.

**1.6 Basic Criteria for Positive Results**

Two core filtering rules are implemented to reduce false positive signals. Sequences derived from phylogenetically closely related microorganisms are eliminated to avoid cross-species misidentification. In addition, any microbial species detected in nucleic acid extraction negative controls are excluded from candidate positive results. All remaining candidate pathogens require final expert review before being reported as positive findings.

2. **Quantitative Positivity Thresholds for the NTS Assay**

A full set of quantitative thresholds, optimized via receiver operating characteristic (ROC) curve analysis, are established to distinguish true pathogens from background noise and contamination.

First, strict quality thresholds are applied to sequencing reads. Only reads with a Phred quality score of 7 or above and a length between 200 and 2000 nucleotides are retained for downstream analysis. For reads aligned to bacterial 16S rDNA and fungal ITS1/2 regions, a minimum alignment coverage of 90% is required.

Reads Per Million (RPM) is adopted as the indicator of microbial relative abundance. To eliminate interference from closely related microbial taxa, abundance correction is performed: the RPM value of microorganisms belonging to the same genus is reduced by 10%, and the RPM value of organisms from the same family but different genera is reduced by 5%. Any taxa with an adjusted RPM of zero after correction are ruled out as non-target interference.

Multiple fold change thresholds relative to different negative controls are defined for final positive judgment. For bacterial detection, a sample is deemed positive when the long-term negative control fold change (LNC-FC) exceeds 23.299 and the dynamic negative control fold change (DNC-FC) exceeds 25.493. For fungal detection, the corresponding cutoffs are set at 0.849 for LNC-FC and 1.666 for DNC-FC. Microbial species detected in over 50% of no-template control (NTC) samples are classified as universal PCR contaminants and excluded from all test results.

Prior to threshold calculation, all samples are normalized to a unified sequencing depth of 10,000 valid reads to guarantee consistent comparison across all specimens.

Bacteria (not on the critical pathogen list): Positive if (a) species‑level mapped reads in the sample >100, or (b) the microbe has the highest mapped read count among all detected microorganisms in the sample, and the ratio of sample reads to negative‑control reads is >10.

Fungi (not on the critical pathogen list): Positive if (a) species‑level mapped reads >20, or (b) relative abundance >50%, and the ratio of sample reads to negative‑control reads is >10.

Pathogens on the critical list (e.g., Mycobacterium tuberculosis, Nocardia spp.): Positive if species‑level mapped reads >2, regardless of relative abundance, due to their high clinical significance and low background prevalence.

Coverage requirement: Genomic coverage (e.g., base‑coverage proportion or depth) was not used as an additional criterion for determining positivity in this study. All positive calls were based solely on the read‑count and ratio thresholds specified above.

Confidence score: No bioinformatics‑derived confidence score was applied as an independent filter. Instead, the interpretation of the culture and sequencing results was based on the types of samples and microbes detected in the samples. The final report for each sample was discussed with a clinical microbiology specialist and clinicians responsible for each patient on an individual basis. In brief, for culture, any clone found on the plate was defined as a positive result in specimens from sterile settings and reported to the clinicians after eliminating the possibility of contamination. For specimens from non-sterile settings, the type and quantity of strains were both considered for judgment of positive results according to the ESCMID guidelines.

Critical pathogen list: A reportable list of clinical pathogens was set up referred to published papers which apply next generation sequencing to identify the pathogens in sepsis (https://kariusdx.com/karius-test/pathogens).

3. **Negative Control Setup and Background Signal Management**

All raw negative‑control data are provided in Supplementary material 3. Low‑level reads from environmental or commensal microorganisms, including Halomonas mongoliensis, Sphingomonas oligophenolica, Delftia acidovorans, e.g. , were observed, with mean species‑level read counts ranging from 0 to 772 reads.

**3.1 Organisms detected in negative controls and their occurrence frequency:**

In our negative controls (including ETC, NTC, BatchNC, Long‑termNC and DynamicNC), the top 20 frequently detected microorganisms were environmental or human commensal flora, including:

| taxonomy | Frequency | Mean(reads) | Q1(reads) | Median(reads) | Q3(reads) |
| --- | --- | --- | --- | --- | --- |
| Halomonas mongoliensis | 0.51 | 507 | 0 | 49 | 429 |
| Sphingomonas oligophenolica | 0.45 | 772 | 0 | 51 | 476 |
| Delftia acidovorans | 0.43 | 246 | 0 | 25 | 200 |
| Sphingomonas mali | 0.42 | 53 | 0 | 4 | 32 |
| Sphingomonas insulae | 0.41 | 62 | 0 | 3 | 27 |
| Halomonas socia | 0.39 | 57 | 0 | 3 | 48 |
| Halomonas stevensii | 0.38 | 90 | 0 | 2 | 51 |
| Halomonas pantelleriensis | 0.36 | 20 | 0 | 1 | 17 |
| Halomonas saccharevitans | 0.36 | 18 | 0 | 1 | 15 |
| Halomonas hamiltonii | 0.36 | 63 | 0 | 1 | 36 |
| Halomonas campaniensis | 0.36 | 38 | 0 | 1 | 25 |
| Sphingomonas mucosissima | 0.35 | 12 | 0 | 1 | 7 |
| Halomonas shengliensis | 0.35 | 133 | 0 | 1 | 67 |
| Aquabacterium parvum | 0.33 | 230 | 0 | 0 | 146 |
| Halomonas meridiana | 0.33 | 87 | 0 | 0 | 46 |
| Sphingomonas aquatilis | 0.32 | 11 | 0 | 0 | 4 |
| Sphingomonas echinoides | 0.31 | 5 | 0 | 0 | 3 |
| Halomonas ventosae | 0.30 | 13 | 0 | 0 | 7 |
| Halomonas axialensis | 0.30 | 31 | 0 | 0 | 8 |
| Aquabacterium fontiphilum | 0.29 | 25 | 0 | 0 | 18 |
| Halomonas boliviensis | 0.29 | 26 | 0 | 0 | 6 |

All raw detection data for each type of negative control are now provided in Supplementary material 3.

**3.2 Multi‑layer bioinformatic filtering strategy for background signal elimination (Figrure S1):**

Direct exclusion layer: All microbial species detected in ETC, NTC and BatchNC were directly excluded.

Fold‑change layer: A candidate microorganism was retained only when its abundance fold changes against Long‑termNC, DynamicNC and BatchNC simultaneously exceeded the pre‑optimised thresholds.

The specific criteria were as follows:

For bacterial detection: LNC‑FC > 23.299, DNC‑FC > 25.493, and BNC‑FC > 5.

For fungal detection: LNC‑FC > 0.849, DNC‑FC > 1.666, and BNC‑FC > 5.

These thresholds were optimised by ROC analysis as described in our previous work [citation: Hong et al., 2023, DOI: 10.1111/jcmm.17651].

Frequency‑filtering layer: Microbes with a detection frequency >50% in NTCs were removed as widespread PCR contaminants.

Closely‑related taxa correction: Abundance correction for closely related microbial taxa was performed to reduce noise from homologous sequence alignment.

Clinical review layer: All remaining candidate pathogens were evaluated by clinical microbiologists and physicians in combination with patients’ clinical manifestations to eliminate residual background interference.

**3.3 Other control measures (extraction blanks, NTCs, sequencing controls, batch processing, carryover prevention):**

These details are now comprehensively described in the revised Methods section:

Extraction blanks: Tris‑EDTA buffer processed in parallel with clinical samples through the entire lysis, purification and elution workflow.

No‑template controls: Two NTCs per sequencing library to monitor PCR reagent contamination.

Sequencing controls: BatchNC to track batch‑specific background; all runs used the same reagent lots, R9.4.1 flow cells and GridION X5 platform; datasets normalised to 10,000 valid reads.

Batch processing: Each library contained clinical samples plus two ETCs and two NTCs; 16S and ITS products pooled at 10:3 ratio.

Carryover contamination prevention: Strict laboratory zoning (separate areas for extraction, PCR, library preparation and sequencing); UV‑sterilised laminar flow hood; 10% bleach and 75% ethanol surface decontamination before each experiment; disposable filter‑tips and consumables; air‑settling plates for environmental monitoring; synchronous manipulation of samples and controls to minimise exposure time.

4. **Sample Manipulation, Batch Processing and Contamination Control Measures**

**4.1 Sample handling:**

For EDTA-anticoagulated whole blood samples, a two‑step centrifugation protocol was applied: samples were first centrifuged at 800 × g for 10 minutes at room temperature, after which red blood cells were discarded and the supernatant containing plasma and leukocytes was collected; a second centrifugation at 16,000 × g for 10 minutes was performed, and the final precipitate was used for nucleic acid extraction. For ascitic fluid specimens, samples were mixed with 0.1 mm glass beads and vortexed at 3000 rpm for 5 minutes to mechanically disrupt microbial cell walls and release nucleic acids. All samples were processed immediately after collection to prevent nucleic acid degradation and unintended microbial growth.

**4.2 Batch processing:**

Amplified products targeting 16S rRNA and ITS1/2 from the same sample were pooled at a fixed mass ratio of 10:3. Mixed products from different individual samples were combined equally to construct unified sequencing libraries. Each library was configured with clinical samples plus two ETCs and two NTCs, ensuring synchronous monitoring of contamination for the entire batch.

**4.3 Extraction controls:**

Extraction negative controls were prepared using Tris-EDTA buffer instead of clinical samples and underwent exactly the same lysis, purification and elution procedures as test samples in each batch, accurately reflecting contamination risks during the nucleic acid extraction step.

**4.4 Sequencing batch-effect control:**

All sequencing experiments were conducted under standardised experimental conditions to minimise technical variation. The same reagent batches, R9.4.1 flow cells and GridION X5 sequencing platform were used across all runs. Batch‑specific background signals were corrected using fold‑change values calculated from BatchNC. In addition, all sequencing datasets from different batches were normalised to a unified depth of 10,000 valid reads to eliminate deviations caused by uneven sequencing output.

**4.5 Environmental contamination control:**

Contamination control was implemented through combined physical operational regulations and bioinformatic filtering measures.

At the laboratory operation level: Strict zoning was enforced – independent work areas were designated for nucleic acid extraction, PCR amplification, library preparation and on‑machine sequencing to avoid aerosol cross‑contamination. A full set of negative controls covering every experimental stage (ETC, NTC, BatchNC, Long‑termNC, DynamicNC) was deployed to capture persistent, transient and batch‑specific environmental microbes. Disposable consumables, including pipette tips, centrifuge tubes and lysis tubes, were used for each sample to avoid cross‑contamination, and common reagents were prepared in uniform batches. Samples and controls were manipulated synchronously throughout lysis and purification to shorten exposure time in the laboratory environment. In addition, nucleic acid extraction and PCR setup were performed in a dedicated clean room under a laminar flow hood with UV sterilisation. Work surfaces and environmental surfaces were cleaned with 10% bleach and 75% ethanol before each experiment, and air-settling plates were routinely placed in the laboratory to monitor airborne microbial load.

At the bioinformatic filtering level: Microbial sequences matching flora identified in Long‑termNC were removed as persistent laboratory environmental contaminants. Microbes detected in DynamicNC were filtered out as temporary environmental pollutants. All taxa found in BatchNC were directly eliminated as batch‑related contamination. Combined with microbial profiles from healthy human samples, human commensal flora were distinguished from true pathogens. Ultimately, only microorganisms with abundance significantly higher than all negative‑control baselines were retained as candidate infectious pathogens.

5. **Rationale for Sample Fraction Selection and Its Impact on Pathogen Detection**

Plasma combined with leukocyte fractions are selected for testing instead of whole blood primarily to mitigate interference from host genomic DNA. Whole blood contains massive amounts of human DNA derived from blood cells, which competes with microbial nucleic acids during PCR amplification and sequencing, and substantially reduces the detection sensitivity of the NTS platform. Removing red blood cells and retaining plasma and leukocytes effectively reduces human DNA background. Meanwhile, this fraction can capture both extracellular pathogens and cell-free microbial DNA present in plasma, as well as intracellular pathogens residing within leukocytes, covering the main forms of pathogens in blood samples. This pretreatment strategy is also well adapted to the characteristics of Nanopore targeted sequencing, which has limited tolerance for samples with extremely high host DNA content, and balances detection performance and testing cost effectively.

This sample selection exerts different impacts on the detection of various pathogens. For intracellular organisms, leukocytes carrying intracellular bacteria and fungi are fully preserved. Mechanical lysis during nucleic acid extraction thoroughly releases intracellular microbial DNA, so the detection efficiency for intracellular pathogens is not compromised, and is even improved compared with using pure plasma alone.

For fungi, fungal spores and vegetative cells exist either as free particles in plasma or are phagocytosed by leukocytes. The plasma-leukocyte fraction covers both distribution forms of fungi. Combined with high-specificity fungal ITS primers, the overall detection performance for fungi remains stable, and multiple fungal species are successfully identified in this study.

For low-burden bacteremia, removing abundant human DNA increases the relative proportion of low-abundance pathogen nucleic acids, making low-copy microbes easier to be amplified and detected. Although a tiny quantity of trace pathogens may be lost during centrifugation for extremely low-burden samples, this limitation is offset by multiplex PCR enrichment and optimized abundance thresholds. The platform still achieves reliable detection for low-burden bacteremia cases.

6. **Workflow Turnaround Time Breakdown and Clinical Practical Feasibility**

The entire NTS workflow is divided into sequential steps with defined durations, and the detailed time breakdown is as follows:

6.1 Sample pre-treatment and nucleic acid extraction: approximately 60 minutes, including sample mixing, mechanical lysis and column-based purification, with negative controls processed in parallel.

6.2 First-round multiplex target PCR: approximately 120 minutes for amplifying bacterial and fungal target fragments.

6.3 Second-round barcode PCR: approximately 30 minutes to add barcode sequences for sample differentiation.

6.4 Amplicon pooling and adapter ligation for library preparation: approximately 30 minutes to complete final library construction.

6.5 On-platform sequencing: ranging from 10 minutes to 360 minutes; a short sequencing run of 10 minutes is applied for emergency cases, while 3 to 6 hours of sequencing is adopted for routine tests.

6.6 Bioinformatic analysis: approximately 10 minutes via automated analysis pipelines to generate preliminary microbial identification results.

6.7 Expert review and final report generation: less than 60 minutes for professional verification and formal report issuance.

In terms of total turnaround time, the shortest total duration for all experimental and analytical steps is 4.5 hours when using the emergency short sequencing protocol, and a preliminary report can be released within 6 hours for urgent clinical scenarios. For routine testing with full-length sequencing, the total time of the complete experimental workflow ranges from 6 to 11 hours. From sample collection to the release of the final formal clinical report, the overall turnaround time for all specimens is controlled within 19 hours.

This workflow is highly feasible for routine operation in clinical laboratories. Most core steps including PCR amplification, sequencing and bioinformatic analysis are fully automated by dedicated instruments, which minimizes manual labor. Multiple batches of samples can be processed in parallel during extraction and PCR procedures, making the platform suitable for high-throughput clinical testing. The flexible sequencing duration can be adjusted according to clinical demands: shortened sequencing meets the requirements of emergency diagnosis, while extended sequencing ensures sufficient data depth for routine samples. This design fully adapts to the differentiated testing needs of clinical laboratories.
